# An evaluation of the validity of an in vitro and an in situ/in vitro procedure for assessing protein digestibility of blood meal, feather meal and a rumen-protected lysine prototype

**DOI:** 10.1093/tas/txac039

**Published:** 2022-04-02

**Authors:** Kari A Estes, Peter S Yoder, Clayton M Stoffel, Mark D Hanigan

**Affiliations:** 1 Department of Dairy Science, Virginia Polytechnic Institute and State University, Blacksburg, VA 24061, USA; 2 Balchem Corporation, New Hampton, NY 10958, USA; 3 Perdue AgriBusiness LLC, Salisbury, MD 21804, USA; 4 Papillon Agricultural Company, Easton, MD 21601, USA

**Keywords:** digestibility, in vitro, protein

## Abstract

In vitro procedures are commonly used to estimate rumen protein degradability and protein digestibility of feed ingredients. However, it is unclear how well these assays correlate to in vivo data. The objectives of this work were to compare postruminal protein availability estimates from one in vitro procedure and one in situ/in vitro procedure with in vivo observations for blood meal (BM), feather meal (FM), and a rumen-protected lysine prototype (RP-Lys). The FM and BM used for this experiment were subsamples of material assessed in vivo by an isotope-based method and the RP-Lys subsamples were of a prototype tested in two in vivo trials: a lactation trial and by plasma appearance. Subsamples of the BM (*n* = 14) and the FM (*n* = 22) were sent to each of three different laboratories for in vitro or in situ/in vitro analysis of crude protein (CP) and determination of rumen undegraded protein (RUP) and digested RUP (dRUP). Subsamples of the RP-Lys (*n* = 5) were sent to one laboratory for in vitro analysis of CP, RUP, and dRUP. Two diets containing BM or FM were assessed using the Cornell Net Carbohydrate and Protein System (CNCPS) v6.55 with ingredient inputs derived from either the CNCPS feed library, the isotope dilution method, or an average of the in vitro results from the three laboratories to determine how much the differences among estimates affected ingredient values. In vitro dRUP estimates for BM from one laboratory closely matched those determined in vivo (66.7% vs. 61.2%, respectively), but no in vitro estimates for FM matched the in vivo values. Surprisingly, there were significant differences in protein digestibility estimates from the modified three-step procedure across the two laboratories for BM (*P* < 0.0001) and for FM (*P* < 0.0001) indicating significant variation among laboratories in application of the method. Within all laboratories, BM estimates were reported in a narrow range (CV values of 2.6 or less). However, when testing multiple samples of FM or the RP-Lys prototype, CV values within a laboratory ranged up to 11 and 34, respectively. For the RP-Lys, dRUP estimates from the in vitro method were roughly half of that determined by the in vivo methods suggesting poor concordance between the in vitro and in vivo procedures for this ingredient. The inconsistencies within and among laboratories accompanied with dissimilarities to in vivo data is problematic for application in nutrition models. Additional refinement to the in vitro techniques is warranted.

## INTRODUCTION

Dietary protein represents a significant economic proportion of lactating cow diets, and it is used inefficiently under current feeding management regimes (Huhtanen and Hristov, 2009). This inefficiency leads to significant environmental nitrogen loading and lost economic opportunity (Agle et al., 2010). To achieve optimal efficiency, diets must be optimized for rumen degraded protein (RDP) supply to support microbial growth in the rumen, and postruminal digestible amino acid (AA) supplies to match animal needs (NRC, 2001). This requires precise knowledge of AA supply and use. The former is challenging due to the range of ingredients fed and the variability in nutrient composition of those ingredients over time and source. Thus, robust methods of assessing RDP and intestinally digestible AA supplies that can be conducted rapidly, with good precision and accuracy, and for a reasonable cost are required.

In situ methods have served as the basis for ruminal outflow estimates (Orskov and McDonald, 1979) and have been used to estimate intestinal digestion (Jarosz et al., 1994). However, the method requires surgically altered animals and the analyses cannot be conducted rapidly. Additional limitations include the loss of small particles from the bags that are not truly solubilized, microbial contamination, potentially impaired movement of enzymes and microbes into the bag (e.g., bag pore size) (Stern et al., 1997), and the assumption that all soluble protein is degraded (White et al., 2017). The extent of ruminal digestion also requires assumptions or estimates of the ruminal residence time to calculate passage rate (kp), and these estimates have been shown to be severely biased (White et al. 2017). The mobile bag method for assessment of postruminal digestibility may also overestimate digestibility if collected in feces due to large intestine fermentation and residence times that often exceed normal intestinal residence times (e.g., 42 h) (Arriola Apelo et al., 2014).

In vitro methods are attractive because of speed and cost. The primary in-vitro methods available for measuring intestinal digestibility include the Minnesota three-step procedure (Calsamiglia and Stern, 1995), the modified three-step (Gargallo et al., 2006), and a revised three-step method (Ross et al., 2013). All three methods attempt to simulate ruminal, abomasal, and intestinal processes. However, they differ in enzyme types and concentrations, incubation times, methods of termination, and equipment. These methods have been found to properly rank feed ingredients relative to protein availability (Calsamiglia and Stern, 1995; Stern et al., 1997; Gargallo et al., 2006; Ross et al., 2013), and the rankings correlated with in vivo performance (Noftsger and St-Pierre, 2003; Gutierrez-Botero et al., 2014). However, the methods have not been compared to the absolute values obtained from in vivo measurements. There is also variation among and within laboratories introduced by variation in the enzymes used to replicate digestion (Stern et al., 1997). Ammonia accumulation in batch cultures can also be problematic (Colombini et al., 2011) causing rates of degradation estimates to decline over the incubation time (Paz et al., 2014). The original three-step procedure (Calsamiglia and Stern, 1995) used to replicate ruminal and intestinal digestion also lacks the ability to determine individual AA digestibility due to termination of incubations with trichloroacetic acid (Ross et al., 2013).

The revised three-step procedure by Ross and colleagues (2013) is composed of a 16 h incubation in rumen fluid followed by a 1 h incubation in pepsin and then a 24 h incubation in trypsin, chymotrypsin, amylase, and lipase. Though this technique has not yet been published in a peer reviewed journal, this procedure has been adopted by commercial laboratories to determine ingredient digestibility and is being used by industry professionals in ration formulation software and by companies to set bioavailability values for rumen protected ingredients (Diaz et al., 2018 [abstract]; Diaz et al., 2020 [abstract]). However, how these results compare to absolute values obtained from in vivo measurements is yet to be determined.

The objectives of this work were to compare two commercially available in vitro procedures (a pepsin digestibility assay and a revised three-step procedure) to in vivo observations of one source each of blood meal (BM) and feather meal (FM). A secondary objective was to compare the revised three-step procedure to in vivo observations for a rumen-protected lysine prototype (RP-Lys).

## MATERIAL AND METHODS

Nutrient digestibility and availability is defined herein as the proportion of nutrient that is accessible for biological use by the animal.

The BM and FM used in this experiment were subsamples of the same material assessed by Estes et al. (2018) using an in vivo, stable isotope-based approach. The BM was of porcine origin and was ring dried. The hydrolyzed FM was from a chicken processing facility where feathers from slaughtered poultry were treated under pressure and later ground. The FM used in this experiment did contain coagulated poultry blood. The RP-Lys prototype (an encapsulate consisting of an AA core and lipid coating manufactured by Balchem Corporation, New Hampton, NY) was the ingredient referred to as P4 in the lactation response trial and plasma appearance trial reported by Fleming et al. (2019).

To ensure that the laboratory results were representative of the expected mean and that potential differences among laboratory could be detected, multiple subsamples of each ingredient were submitted for analyses. A power test was used to estimate the number of BM and FM samples to submit. The use of cecectomized roosters to determine small intestine digestibility of amino acids (specifically for BM, fish meal, soybean meal, and corn gluten meal) has previously been validated using a bovine in vivo method using duodenal and ileal flows in cannulated cattle (Titgemeyer et al., 1990). Thus, total tract digestibility data from cecectomized roosters were used for the expected mean, and the variance was derived from in vitro results from four commercial laboratories testing three batches of BM and FM. The tests indicated that detecting a 10% difference in total tract digestibility at a power of 0.80 required 22 samples of FM and 14 samples of BM per laboratory. The retained RP-Lys from the Fleming et al. (2019) trial limited testing to five subsamples for a single location.

All samples were analyzed for crude protein (CP), rumen undegraded protein (RUP), and digested RUP (dRUP). For BM and FM, subsamples (462.2 ± 26.4 g; *n* = 22 for FM and *n* = 14 for BM) were collected from the material used for the in vivo trials, divided into thirds, with one third submitted to each of the three laboratories. 

The laboratories that conducted the analysis were denoted as Lab1, Lab2, and Lab3. All three reported dry matter (DM; samples dried at 60 °C in a forced-air oven), CP (method 990.03; AOAC International, 2012), RUP, and intestinally digested protein (IDP). Lab1 also reported soluble protein (samples incubated in water for one hour and filtered on 2.7 micron filter), RDP, and total tract CP digestibility. Lab2 reported RDP (calculated as [100-RUP]) and total tract undigested CP (calculated as [100 − (RDP + IDP)]). IDP is denoted herein as dRUP (%CP). Each laboratory conducted a single analysis of each subsample per measurement. Table 1 lists the assays performed at each laboratory. Lab1 and Lab2 used the revised three-step procedure developed by Ross and colleagues (2013) to estimate RUP and dRUP. However, Lab1 did make several modifications to the technique: (1) pure cellulose was used for the blanks instead of neutral detergent treated corn silage due to consistency and handling ease, (2) 2.7 micron filters were utilized instead of the recommended 1.5 micron filters, (3) hot water (54–60 °C) was used to terminate the assay instead of boiling water, and (4) bile salts were added to the 24 hour intestinal incubation step containing trypsin, chymotrypsin, amylase and lipase. Similarly, both Lab1 and Lab2 determined nitrogen content of the residues via combustion analysis instead of Kjeldahl. Lab3 used a 16 h rumen in situ incubation coupled with a pepsin digestibility assay (method 971.09; AOAC International, 2012) to estimate RUP and dRUP. In brief, the 16 h rumen in situ technique used rumen cannulated lactating cows fed a typical Northeastern high cow TMR. The method consisted of 5 g of ingredient DM heat sealed into 10 × 20 cm dacron bags (59 micron pore size, Ankom Inc., Fairport, NY) positioned under the rumen mat and incubated for 16 h. The assay was conducted in triplicate. Upon removal, all bags were hand washed under cool running water until the rinse water became clear and dried in a convection oven at 2 °C for a minimum of 48 h. Dried residue and a subsample of the source material were analyzed for DM and CP content. Ingredient RUP was estimated as the proportion of the initial ingredient CP that remained after 16 h of incubation. The aforementioned analyses were chosen because they were standard procedures available at each of the laboratories. Though not a primary method used in the industry today, the pepsin digestibility assay utilized by Lab3 was included in this experiment because of pepsin’s active role in cleaving proteins into soluble peptides. Qiao and colleagues (2004) found that an exhaustive in vitro procedure (0.25% pepsin concentrated solution incubated for 24 h coupled with a 96 h intestinal digestion) still did not yield the small peptide sizes observed at the ileum in pigs, suggesting that perhaps the enzyme concentrations and incubation times used in the method developed by Ross et al. (2013) may not be adequate. The pepsin digestibility assay from Lab3 used a higher concentration of pepsin (0.2%) and longer incubation time (16 h) than those in the revised three-step procedure.

**Table 1.** Methods used to estimate protein digestibility from three commercial laboratories

Commercial laboratory	Reference method	Assay details	
		RUP^1^	dRUP^2^
Lab1	Revised three-step procedure developed by Ross and colleagues (2013)	16 h in vitro incubation in rumen fluid	1 h in vitro incubation in pepsin + 24 h in vitro incubation in trypsin, chymotrypsin, amylase, lipase and bile salts
Lab2	Revised three-step procedure developed by Ross and colleagues (2013)	16 h in vitro incubation in rumen fluid	1 h in vitro incubation in pepsin + 24 h in vitro incubation in trypsin, chymotrypsin, amylase and lipase
Lab3	16 h rumen in situ incubation coupled with a pepsin digestibility assay (method 971.09; AOAC International, 2012)	16 h in situ rumen incubation	16 h in vitro incubation in pepsin

Rumen undegradable protein.

Digestible rumen undegradable protein.

The RP-Lys prototype was subsequently sent to Lab1 and assessed for DM, CP, soluble protein, RUP, dRUP and total tract digestibility analysis. Lab1 was chosen because it provided results from the prior work that most closely replicated in vivo results. For the RP-Lys prototype, the final step of the procedure developed by Ross and colleagues (2013) was modified to recover material after freeze drying instead of hot water to accommodate the lipid coating of the prototype.

RUP digestibility coefficients (DC_RUP_) were calculated from in vitro tests using RUP and dRUP estimates:


$$\begin{array}{l} DC_{RUP}=\;\left[dRUP\;\left(\%\;of\;CP\right)/RUP\;\left(\%\;of\;CP\right)\right] \end{array}$$

The effect of laboratory on in vitro protein digestibility was tested using the GLM procedure of SAS 9.3, and the model:


$$\begin{array}{l} y_{i}=\mu +\alpha_{i}+\varepsilon_{i} \end{array}$$

where *y*_*i*_ = the dependent variable, μ = the mean response, α_i_ = the effect of laboratory, and ε_i_ = the residual errors. Laboratory was considered a fixed effect. Post-test comparisons of the means were made using the LSMEANS statement in SAS with the HSD option. For all tests, *P* values less than 0.05 were considered significant and *P* values greater than 0.05 and less than 0.10 were considered trends.

The NDS Professional ration formulation software (version 6.55; RUM&N, NDS Professional, Reggio Nell’Emilia, Emilia-Romagna, Italy) was used to evaluate a diet containing BM or FM using ingredient inputs derived from one of three sources: (1) CNCPS feed library values, (2) the isotope method (Estes et al., 2018), or (3) an average of the in vitro results from the three laboratories (however, soluble protein was only reported by Lab1 and was thus the only value used for that particular input). The CNCPS ingredient specification was constructed to yield the average RUP and dRUP from the three labs, not necessarily average of individual pools and degradation rates, which were adjusted to yield the average RUP and dRUP. Hence, having only Lab1 report soluble protein is of minimal concern since the objective is for spec that yields the average RUP and dRUP. For the CNCPS protein pool inputs, the A1 ammonia pool was set to zero and the A2 true soluble protein pool set to equal the measured soluble protein. The C pool, which represents indigestible protein, was set equal to the product of the laboratory or isotope assay measured dRUP and DC_RUP_. The B2 pool was set to zero, because, by biological definition, animal protein ingredients should not contain fiber bound protein. The B1 pool was calculated by subtraction of A1, A2, B2, and C pools from CP. The first diet (Diet1) was that from Estes et al. (2018) which contained 69.2% forage with a predicted forage passage rate of 1.42% per h, concentrate passage rate of 4.57% per h, and liquid passage rate of 8.18% per h, for an animal consuming 7.80 kg DM/d and weighing 340 ± 34 kg. This represented an energy intake of 1.9× maintenance. The second diet (Diet2) contained 50% forage with a predicted forage passage rate of 1.50% per h, concentrate passage rate of 5.79% per h, and liquid passage rate of 11.55% per h, for an animal consuming 21.14 kg DM/d (3× maintenance) and weighing 708 kg. The isotope-based results were set to equal the results published by Estes et al. (2018) using the research diet (Diet1), animal description, and observed intake. The input CNCPS rates (i.e., *kd* [ruminal degradation rate for each protein fraction (%/h)]) were adjusted to yield RUP, dRUP and DC_RUP_ similar to the observation of Estes et al. (2018) for the isotope-based results for Diet1 and average of the three laboratory results for Diet2.

## RESULTS AND DISCUSSION

The mean, minimum, and maximum values, and CV for analyses of CP, RUP, and dRUP for BM and FM are shown in Table 2. The mean, minimum, and maximum values, and CV for analyses of CP, RUP, and dRUP by Lab1 for the RP-Lys prototype are shown in Table 3.

**Table 2.** Variation in ring-dried blood meal and hydrolyzed feather meal (with blood) within and across laboratories

Variable	LSMean	SE	Minimum	Maximum	CV
Blood meal^1^:					
CP%, %DM					
Lab1	101.8^a^	0.3	101.2	102.7	0.5
Lab2	101.2^a,b^	0.3	95.2	102.7	2.0
Lab3	100.6^b^	0.3	100.5	100.8	0.1
RUP, %CP^3^					
Lab1	90.3^a^	0.5	88.3	92.6	1.8
Lab2	93.9^b^	0.5	90.9	100.0	2.4
Lab3	96.1^c^	0.5	92.4	98.9	2.1
dRUP, %CP^4^					
Lab1	66.7^a^	0.5	63.6	70.1	2.4
Lab2	93.2^b^	0.5	90.9	99.4	2.4
Lab3	92.4^b^	0.5	88.3	95.3	2.3
DC_RUP_, %RUP^5^					
Lab1	73.9^a^	0.3	70.6	78.0	2.6
Lab2	99.2^b^	0.3	99.0	100.0	0.2
Lab3	96.1^c^	0.3	95.6	96.6	0.2
Feather meal^2^: CP%, %DM					
Lab1	90.8^a^	0.1	89.8	92.5	0.6
Lab2	89.9^b^	0.1	88.2	90.6	0.7
Lab3	89.9^b^	0.1	89.4	90.4	0.3
RUP, %CP					
Lab1	83.2^a^	0.2	81.8	84.7	0.9
Lab2	87.5^b^	0.2	85.6	90.0	1.4
Lab3	90.1^c^	0.2	88.8	94.1	1.4
dRUP, %CP					
Lab1	40.1^a^	0.6	36.8	42.5	3.6
Lab2	33.6^b^	0.6	26.0	41.6	11.8
Lab3	73.3^c^	0.6	71.4	79.0	2.9
DC_RUP_, %RUP					
Lab1	48.1^a^	0.6	44.9	50.5	3.2
Lab2	38.4^b^	0.6	30.2	46.5	11.2
Lab3	81.3^c^	0.6	79.8	84.7	1.9

*n* = 14 samples analyzed per laboratory.

*n* = 22 samples analyzed per laboratory.

Rumen undegradable protein as a percent of crude protein.

Digestible rumen undegradable protein as a percent of crude protein.

Rumen undegradable protein digestibility coefficient (dRUP/RUP × 100).

= Values with differing subscripts within the LSMean column for each specific ingredient variable (CP, RUP, dRUP and DC_RUP_) across laboratories are considered significantly different (*P* < 0.05) using Tukey adjusted comparisons.

**Table 3.** Variation in a rumen-protected lysine prototype^1^* within one laboratory (Lab1)

Variable	Mean	SE	Minimum	Maximum	CV
CP%, %DM	50.8	1.0	47.2	52.9	4.2
RUP, %CP^2^	91.1	0.7	88.8	93.2	1.8
dRUP, %CP^3^	24.3	3.7	10.8	32.7	34.3
DC_RUP_, %RUP^4^	26.6	4.0	12.2	35.9	33.7

*Manufactured by Balchem Corporation, New Hampton, NY.

*n* = 5 samples analyzed in one run.

Rumen undegradable protein as a percent of crude protein.

Digestible rumen undegradable protein as a percent of crude protein.

Rumen undegradable protein digestibility coefficient (dRUP/RUP × 100).

Mean CP values for BM and FM differed across laboratories with Lab1 generally being the greatest and Lab3 the least; however, the differences were very small (differences of 1.2 percentage units and 0.9 percentage units for BM and FM, respectively). The RUP content of BM and FM also differed across laboratories with Lab1 values being the least and Lab3 values being the most; these differences were 6 percentage units for BM and 7 percentage units for FM; however, the in vitro methods of Lab1 and Lab2 were relatively consistent with the in situ procedure used by Lab3. Ruminal protection of the RP-Lys prototype was found to be high both in vitro (Lab1) and in situ (Fleming et al., 2019), suggesting the in vitro method replicated the in situ observations for this type of product.

Lab1 (16 h in vitro incubation in rumen fluid), Lab2 (16 h in vitro incubation in rumen fluid), and Lab3 (16 h in situ incubation) reported seemingly high RUP values for BM. Estes et al. (2018) reported 78.3% of CP determined by a 12 h rumen in situ incubation, the NRC (2001) feed library lists a mean value of 76.5% of CP for ring dried BM, and the NASEM (2021) lists a value of 72.0% of CP for a high dRUP BM, both of which were determined from data published using the rumen in situ methodology. The FM RUP value as determined by 12 h rumen incubation averaged 81.9% of CP (Estes et al., 2018) which was similar to those determined by Lab1 (83.2% of CP) and numerically less than values reported by Lab2 (87.5% of CP) and Lab3 (90.1% of CP). Neither the in situ estimate nor the laboratory estimates closely matched the NRC (2001) reported value for hydrolyzed FM RUP of 63.8% of CP or the NASEM (2021) value of 70.8% of CP. Differences in RUP content for FM specifically between the 12 h in situ and those reported from Lab3 could be explained by differences in bag dimensions and pore size, the amount of feed being tested, the diets being consumed by the ruminally cannulated animals, and the bag wash method (Mohamed and Chaudhry, 2008).

Mean reported dRUP for BM using the revised three-step procedure were quite different between Lab1 and Lab2 (*P* < 0.0001), and the latter did not differ from that derived using the pepsin-based assay of Lab3 (*P* = 0.5505). Conversely, FM dRUP derived using the revised three-step procedure was numerically more similar between Lab1 and Lab2 (40.1 ± 0.6% of CP and 33.6 ± 0.6% of CP, respectively), but diverged for the pepsin-based assay (*P* < 0.0001). The dRUP estimate for the RP-Lys prototype from Lab1 was low at 24.3 ± 3.7% of CP.

Although there were method modifications, it was still surprising that the dRUP estimates for BM were so divergent between Lab1 and Lab2 (approximately 25 percentage points) as both utilized a similar procedure. Conversely, BM dRUP assessments from Lab2 (93.2 ± 0.5% of CP) were more similar to the pepsin-based method of Lab3 (92.4 ± 0.5% of CP) despite different procedures. Lab1 and Lab2 provided more similar estimates for FM, but they still significantly differed by 7 percentage units.

Given the fairly similar estimates of RUP across laboratories, differences in DC_RUP_ generally reflect dRUP estimates. Lab2 and Lab3 generated DC_RUP_ estimates that were greater than Lab1’s estimate of 73.9 ± 0.3% of RUP. Lab1 and Lab2 DC_RUP_ estimates were more similar for FM at 48.1 ± 0.6% of RUP and 38.4 ± 0.6% of RUP, and Lab3 estimates differed more markedly at 81.3 ± 0.6% of RUP.

The DC_RUP_ estimates for the RP-Lys prototype from Lab1 averaged 26.6 ± 4.0% of RUP. The amount of prototype remaining after completion of the in vivo work was only adequate for evaluation at a single laboratory, and thus we were not able to assess variance among laboratories or methods for this ingredient.

Because all three ingredients were previously assessed for intestinal availability using in vivo techniques, we compared the in vitro results to in vivo derived dRUP estimates for these lots of BM and FM based on plasma AA appearance rates with correction for loss during absorption (Estes et al., 2018) (Table 4). Using a stable isotope-based approach, dRUP of FM and BM were estimated to be 52.6% and 61.2% of CP, respectively. Using a 12-h in situ ruminal incubation to estimate RUP, the DC_RUP_ were calculated as 64% and 78% of RUP. The BM dRUP values from the isotope method were most similar to values from Lab1 (61.2% of CP vs. 66.7% of CP). These estimates also closely matched the dRUP values for BM reported by NRC (2001) and NASEM (2021) (61.2% of CP), both of which were determined from data collected from a mix of published mobile bag/in vitro studies. The other two laboratory estimates of dRUP for BM were, on average, 52% higher than the in vivo measurement. Differences among the DC_RUP_ values followed a similar trend where Lab1 was numerically the most similar to the in vivo observation, followed by Lab3 and then Lab2.

**Table 4.** Protein digestibility of ring dried blood meal, hydrolyzed feather meal (with blood) and a rumen protected lysine prototype estimated by in vitro, in situ and in vivo techniques

Variable	Assay type	RUP, %CP^1^	dRUP, %CP^2^	DC_RUP_, %RUP^3^
Blood meal:				
Lab1	In vitro	90.3	66.7	73.9
Lab2	In vitro	93.9	93.2	99.2
Lab3	In situ & in vitro	96.1	92.4	96.1
Isotope Method^4^	In situ & in vivo	78.3	61.2	78.0
NRC (2001)^5^	In situ/in vitro	76.5	61.2	80.0
NASEM (2021)^6^	In situ/in vitro	72.0	61.2	85.0
Feather meal:				
Lab1	In vitro	83.2	40.1	48.1
Lab2	In vitro	87.5	33.6	38.4
Lab3	In situ & in vitro	90.1	73.3	81.3
Isotope Method	In situ & in vivo	81.9	52.6	64.0
NRC (2001)	In situ/in vitro	63.8	41.5	65.0
NASEM (2021)	In situ/in vitro	70.8	48.1	67.9
RP-Lys^7^:				
Lab1	In vitro	91.1	24.3	26.6
Pulse Dose^8^	In situ & in vivo	106.3	44.7	-
Milk Protein Response^9^	In vivo	-	100.0	-

Rumen undegradable protein as a percent of crude protein.

Digestible rumen undegradable protein as a percent of crude protein.

Rumen undegradable protein digestibility coefficient (dRUP/RUP × 100).

Reported by Estes et al. (2018). RUP was estimated via 12 h in situ and dRUP is estimated via in vivo isotope technique.

NRC (2001). Nutrient requirements of dairy cattle, 7th rev. ed. RUP was determined from data published using the rumen in situ methodology. dRUP was determined from data collected from a mix of published mobile bag/in vitro studies.

NASEM (2021). Nutrient requirements of dairy cattle, 8^th^ rev. ed. RUP was determined from data published using the rumen in situ methodology. dRUP was determined from data collected from a mix of published mobile bag/in vitro studies.

Rumen protected lysine prototype manufactured by Balchem Corporation, New Hampton, NY.

Reported by Fleming et al. (2019). An in situ method was used to estimate RUP fraction while the dRUP fraction was estimated using plasma appearance following an abomasal bolus.

Reported by Fleming et al. (2019). A lactation trial was utilized to determine dRUP in vivo by monitoring changes in milk protein following supplementation.

For FM, there was more divergence between in vivo and in vitro measurements. As with BM, Lab1 values for FM dRUP and DC_RUP_ (40.1% of CP and 48.1% of RUP, respectively) were numerically the most similar to estimates from the in vivo work (52.6% of CP and 64% of RUP, respectively), although dRUP was 12.5 percentage points less than in vivo values which is a substantial difference. Lab1 estimates of FM dRUP were also most closely aligned with the NRC (2001) value of 41.5% of CP but less closely aligned with the NASEM (2021) value of 48.1% of CP. Lab2 and Lab3 reported dRUP values of 33.6% of CP and 73.3% of CP yielding a remarkable range for the same ingredient.

Fleming et al. (2019) found that the bioavailability of the RP-Lys prototype was 44.7% using a pulse-dose method, and 100% based on milk protein responses. They concluded that the pulse dose method likely underestimated bioavailability of the prototype and lipid encapsulated products in general due to the artificial nature of the ruminal incubation, efficacy of delivery of the prototype to the abomasum and potential increases in rates of catabolism following such a large dose of amino acid. Conversely the error of estimate for milk protein responses is quite large, and because it is unlikely the Lys in the protype was completely protected from ruminal degradation and completely released the intestine, the authors concluded the true dRUP likely was somewhere in between 44.0% and 100%. Even the more conservative estimate was more than double the 24.3% estimate derived from the revised 3-step procedure at Lab1.

The three-step procedures were designed to measure protein digestibility; however, with encapsulated AA products, the coating must be digested in the post-ruminal portion of the assay to properly assess lipid encapsulated AA. The three-step procedure of Ross and colleagues (2013) does contain lipase, which releases monoglycerides and free fatty acids. However, the activity of pancreatic lipase requires the presence of co-lipase, bile salts, and calcium (Kimura et al., 1982; Hur et al., 2011), of which co-lipase and calcium are not included in the assay. The lack of inclusion of these necessary components in the in vitro assay may be the reason that the availability of the RP-Lys prototype was underpredicted.

Differences in mean values among laboratories were not due to random within laboratory variation. The CV across analytical procedures for BM did not exceed 2.4% suggesting low procedural variation within each laboratory for that ingredient. Variation for FM was only slightly greater for dRUP and DC_RUP_ from Lab1 and Lab3, but greater for Lab2 at 11.8 and 11.2, respectively. Variation in CP and dRUP contents and the DC_RUP_ of the RP-Lys prototype were greater than expected compared to BM and FM when correcting for the number of samples. Differences in CP are likely indicative of sampling problems. Surprisingly, the standard error for RUP content of the prototype was similar to those of BM and FM RUP when correcting for the number of samples.

The dRUP variability aligns with previous findings by Madsen and Hvelplund (1994) for in vitro and in situ procedures testing rumen degradability of soybean meal, coconut meal, cottonseed meal, barley and fish meal. While variability was observed for the in situ incubations, Madsen and Hvelplund (1994) observed the most variation for in vitro water solubility of CP (CV values ranged from 45% to 75% across test ingredients). Laboratory accounted for 56% of the variation, suggesting that differences in technique were the primary contributor of variance despite following a common protocol. Variation across laboratories observed in the work herein could partially be explained by unintentional differences in sample handling and processing. However, most of this variation, particularly between Lab1 and Lab2 where similar methods were utilized, was likely due to the method modifications made by Lab1, i.e., differences in substrates used for the blanks, filter pore size, use of bile salts and water temperature at the time of assay termination. Variations in RUP fraction across laboratories could be a result of differences in rumen fluid due to animals used and their respective diets, though this has previously been shown to introduce variation to a lesser extent than those mentioned above (Madsen and Hvelplund, 1994). Regardless of the source of interlaboratory variability, the lack of alignment between the in vivo isotope derived observations and in vitro and the variability across laboratories argues against the use of at least the revised three-step procedure as outlined in Ross et al (2013) to derive model input values for field use for BM and FM. Interlaboratory variability could not be determined for the in situ/in vitro combined methodology because only one commercial laboratory performed the procedure, but the potential for this variability still exists. There was also a lack of agreement between the in vivo isotope derived observations and both the RUP in situ and pepsin based dRUP in vitro procedures, suggesting that results from this method should also not be used for model inputs for BM and FM.

To assess the impact of the revised three-step bias for BM and FM, we evaluated a diet containing BM (ring dried) or FM (with blood) using the CNCPS model (v6.55) and ingredient values derived from the CNCPS feed library, the isotope method (Estes et al., 2018) or an average of the in vitro results from the three laboratories (Table 5). The resulting predictions of RUP, dRUP, and DC_RUP_ for two diets (Diet1 and Diet2) are presented in Table 6. The in vitro derived dRUP for FM was underpredicted in both diets. The feed library based dRUP was very close to the in vivo measurement, but this occurred because of a very low estimate of RUP and a high estimate of the DC_RUP_ for FM. The feed library was more closely aligned with in vivo observations for BM with RUP, dRUP, and DC_RUP_ all generally aligning (within 5%), and these values were all less than the in vitro derived estimates regardless of the diet. The in vitro estimates overpredicted RUP and dRUP by roughly 23% and 24%, respectively, when compared to the isotope-based values.

**Table 5.** CNCPS (v6.55) feed library inputs derived from the CNCPS (v6.55) feed library, the isotope measurement method (Estes et al., 2018) or from the average laboratory in vitro results for hydrolyzed feather meal with blood and blood meal (ring dried)

	CNCPS (v6.55) Feed Library			Isotope Assay			Laboratory In Vitro		
	% DM	Kd^1^	Int. Dig^2^	% DM	Kd^1^	Int. Dig^2^	% DM	Kd^1^	Int. Dig^2^
Blood Meal^9^									
CP	95.0	–	–	104.7	–	–	101.2	–	–
Soluble Protein	16.2	–	–	12.1	–	–	10.1^a^	–	–
NDIP^3^	1.3	–	–	13.8	–	–	9.7	–	–
ADIP^4^	1.3	–	–	13.8	–	–	9.7	–	–
CNCPS Pools									
Ammonia, A1	–	200.0	100	–	200.0	100	–	200.0	100
Protein, A2^5^	16.2	11.5	80	12.1	11.5	100	10.1	1.3	100
Protein, B1^6^	77.5	1.10	80	78.8	1.55	100	81.5	0.44	100
Protein, B2^7^	–	–	80	–	–	80	–	–	80
Protein, C^8^	1.3	–	0	13.8	–	0	9.7	–	0
***Feather Meal***									
CP	85.9	–	–	89.0	–	–	90.2	–	–
Soluble Protein	8.6	–	–	11.1	–	–	6.6^a^	–	–
NDIP	1.7	–	–	28.7	–	–	35.9	–	–
ADIP	1.7	–	–	28.7	–	–	35.9	–	–
*CNCPS Pools*									
Ammonia, A1	–	200.0	100	–	200.0	100	–	200.0	100
Protein, A2	8.6	15.6	100	11.1	8.0	100	6.6	8.1	100
Protein, B1	75.6	3.40	100	49.2	0.90	100	47.7	1.39	100
Protein, B2	–	–	80	–	–	80	–	–	80
Protein, C	1.7	–	0	28.7	–	0	35.9	–	0

Ruminal degradation rate percent per hour by protein fraction (%/h).

Intestinal digestibility of protein fractions escaping rumen degradation.

Neutral detergent insoluble protein, not measured, instead, set to equal acid detergent insoluble protein.

Acid detergent insoluble protein, set to equal protein C fraction, indigestible protein.

Protein, A2: True soluble protein (non-ammonia), calculated from soluble protein minus ammonia.

Protein, B1: True insoluble protein, calculated from the difference of CP minus the following pools, protein A1, protein A2, protein B2, and protein C.

Protein, B2: True insoluble protein, calculated from neutral detergent insoluble protein minus acid detergent insoluble protein.

Protein, C: Total tract indigestible protein.

Ring dried.

Value reported is from Lab1 only.

**Table 6.** Prediction of rumen undegradable protein % CP, RUP digestibility %, and absorbed RUP % CP given two simulated diets utilizing the following inputs: CNCPS library inputs, the in vivo isotope assay results, or the laboratory in vitro results

	Feather meal^1^			Blood meal^2^		
	CNCPS	Isotope Assay	In-vitro	CNCPS	Isotope Assay	In-vitro
1.9× Maint^3^						
RUP, %CP^4^	56.3	84.9	84.2	74.5	74.4	91.6
dRUP, %CP^5^	54.3	52.6	44.5	58.5	61.2	82.1
DC_RUP,_ %RUP^6^	96.2	62.0	52.8	78.5	82.3	89.6
3× Maint^7^						
RUP, %CP	62.2	87.6	86.9	78.8	78.6	93.4
dRUP, %CP	60.2	55.4	47.2	61.9	65.4	83.9
DC_RUP_,%RUP)	96.6	63.2	54.3	78.6	83.2	89.8

Feather meal, with blood.

Blood meal, ring dried.

Reported values are predicted by CNCPS v6.55 software with a diet containing 69.2% forage, forage passage rate of 1.42% per hour, concentrate passage rate of 4.57% per hour, and liquid passage rate of 8.18% per hour and an animal with a DMI of 7.80 kg/d and BW of 340 ± 34 kg at 1.9× maintenance (diet is the base diet reported in Estes et al., 2018).

Rumen undegradable protein as a percent of crude protein.

Digestible rumen undegradable protein as a percent of crude protein.

Digestibility of the rumen undegradable protein fraction.

Reported values are predicted by CNCPS v6.55 software with a diet containing 50% forage, forage passage rate of 1.50% per h, concentrate passage rate of 5.79% per hour, and liquid passage rate of 11.55% per hour and an animal with a DMI of 21.14 kg/d at 3× maintenance and 708 kg of BW.

The differences in dRUP between in vivo and in vitro are unlikely to be due to random variations. The in vivo dRUP estimates had standard errors less than 10% of the mean digestible RUP (Estes et al., 2018) versus in vitro standard errors of 0.5% and 0.6% for BM and FM, respectively (Table 2). Thus, the observed deviations between in vitro and in vivo for both ingredients appear to be well outside of the normal variation in the methods. These data suggest that field application of in vitro results without adjustment is problematic and underscores the caution required in application of the methods. Success in application to one ingredient does not ensure applicability for all ingredients. Based on the results reported herein, considerable additional work is required to assess dRUP and AA bioavailability in vivo and compare those results to in vitro methods. In the absence of such work, the application of the in vitro procedures tested herein should be restricted to those ingredients which have been validated and possibly, only for ranking purposes.

The differences among laboratories suggest that there are additional issues to address in standardizing these assays across laboratories and ensuring they are representative of in vivo measurements. Modifications of the three-step procedure by Ross et al. (2013) by Lab1 appear to decrease variability and improve concordance with in vivo data when compared to the unmodified technique utilized by Lab2. But it must be noted that these improvements are not consistent across ingredients or the variable being measured (RUP or dRUP).

Although these in vitro methods appear to be biased, it may still be possible to bias adjust the results for model use if the bias is constant. Such adjustment would require considerable additional in vivo work and will likely differ by ingredient. Despite the bias limitation, the assays may provide a relative ranking within ingredients, i.e., highly digestible vs indigestible BM, FM or rumen-protected AA. But, these relative numbers likely should not be used directly in ration formulation software without scaling. Additionally, one cannot compare across ingredients without scaling, but such scaling factors are not known. Last, given the large variation across laboratories in results, the rankings would only be valid within a laboratory.

In conclusion, the in vitro methods did not reliably represent in vivo values, and they lacked consistency in application across laboratories when evaluating these specific ingredients. Deviations from in vivo were ingredient specific. The range in results was particularly large for FM and the RP-Lys prototype with no laboratory providing assessments that aligned with in vivo results given errors of measurement. In the absence of in vivo validation of the assay for each ingredient, the results from in vitro methods should not be used for model inputs. The application of the in vitro procedures tested herein should be restricted to those ingredients which have been validated and possibly, only for ranking purposes.
